# Concurrent Superior Mesenteric and Splenic Vein Thrombosis in a Prothrombin G20210A Carrier: Radiologic Evolution and Resolution on Apixaban

**DOI:** 10.7759/cureus.106724

**Published:** 2026-04-09

**Authors:** Christopher M Ahmad, Rae-Anne Kastle, Niki Gharavi Alkansari, Phil T Sheridan, Samir Dalia

**Affiliations:** 1 Internal Medicine, Kansas City University, Joplin, USA; 2 Emergency Medicine, Kansas City University, Joplin, USA; 3 Internal Medicine/Hematology, Mercy Hospital Joplin, Joplin, USA

**Keywords:** doac, inherited thrombophilias, prothrombin g20210a mutation, splanchnic venous thrombosis, superior mesenteric and splenic vein thromboses

## Abstract

Splanchnic venous thrombosis (SVT) is an uncommon vascular condition that may occur in association with liver disease, malignancy, or intra-abdominal inflammatory processes. In cases where these conditions are absent, and SVT occurs as a primary disease process, an underlying thrombophilic predisposition should be considered. Concurrent involvement of the superior mesenteric and splenic veins in this context is uncommon and may suggest an inherited prothrombotic tendency. We describe a 76-year-old man with a known heterozygous prothrombin G20210A mutation who presented with several days of abdominal discomfort and was found to have non-occlusive thrombosis of the superior mesenteric vein extending into the splenic vein. Contrast-enhanced computed tomography (CT) confirmed the diagnosis, and a structured evaluation, including positron emission tomography (PET)-CT and comprehensive hypercoagulable testing, excluded malignancy and other acquired causes. Anticoagulation with apixaban was initiated, and follow-up imaging at three months demonstrated complete radiographic resolution. The patient remained clinically stable without bleeding or recurrent thrombosis at six months. This case highlights the importance of systematic etiologic evaluation in non-cirrhotic SVT and illustrates how an inherited thrombophilic predisposition, such as heterozygous prothrombin G20210A mutation, may contribute to thrombosis in the appropriate clinical context.

## Introduction

Splanchnic venous thrombosis (SVT) refers to thrombosis involving the portal, mesenteric, or splenic venous systems and represents a rare vascular disorder, with reported population incidence generally ranging from approximately 0.7 to 3.8 cases per 100,000 persons annually [1-3]. Cirrhosis and intra-abdominal malignancy account for a substantial proportion of cases; however, approximately 20-30% occur in patients without identifiable precipitating factors [4,5].

In non-cirrhotic, non-malignant presentations, inherited thrombophilias warrant consideration. The prothrombin G20210A mutation is one of the most common hereditary hypercoagulable states and is associated with elevated plasma prothrombin levels and an increased lifetime risk of venous thromboembolism [6]. Although well described in deep vein thrombosis and pulmonary embolism, its contribution to thrombosis within the splanchnic circulation is less frequently encountered and is often multifactorial [7].

Thrombosis involving the superior mesenteric and splenic veins carries the risk of bowel ischemia and portal hypertensive complications, underscoring the need for timely diagnosis and appropriate anticoagulation [8]. Emerging observational data support the use of direct oral anticoagulants (DOACs) in selected patients with non-cirrhotic SVT, though randomized data remain limited [9].

We present a case of concurrent superior mesenteric and splenic vein thrombosis in a patient with a heterozygous prothrombin G20210A mutation, highlighting the diagnostic evaluation of non-cirrhotic SVT, the exclusion of secondary provoking factors, and the favorable radiologic course observed during anticoagulation therapy. This report aims to describe the clinical presentation, diagnostic workup, and therapeutic course of thrombophilia-associated SVT in an older adult.

## Case presentation

A 76-year-old man with a medical history of type 2 diabetes mellitus, hypertension, and hyperlipidemia presented in July 2025 with several days of vague abdominal discomfort and early satiety. He denied nausea, vomiting, fever, gastrointestinal bleeding, or recent infectious symptoms. Prior outpatient metabolic testing approximately eight months before presentation demonstrated a total cholesterol of 180 mg/dL, triglycerides of 127 mg/dL, high-density lipoprotein (HDL) cholesterol of 42 mg/dL, low-density lipoprotein (LDL) cholesterol of 113 mg/dL, and non-HDL cholesterol of 138 mg/dL, consistent with chronic cardiometabolic risk factors. His history was notable for a heterozygous prothrombin G20210A mutation, identified approximately eight months prior to presentation during earlier hematologic evaluation, despite no prior personal history of venous thromboembolism. Additional thrombophilia-related testing available from the prior evaluation was likewise obtained approximately eight months before the present thrombotic event. He was a lifelong nonsmoker and reported no alcohol use. On examination, vital signs were stable. Abdominal examination revealed mild epigastric tenderness without guarding, rebound, or peritoneal signs. Laboratory evaluation demonstrated mild anemia without leukocytosis or thrombocytopenia. Renal function was preserved, and lipase was within normal limits (Table 1).

**Table 1:** Baseline laboratory studies obtained at presentation prior to anticoagulation initiation Baseline hematologic and metabolic laboratory studies obtained at presentation. Renal function was preserved. Lipase was within normal limits, and there was no laboratory evidence to suggest pancreatitis at the time of presentation. WBC: white blood cell count; eGFR: estimated glomerular filtration rate; BUN: blood urea nitrogen; µL: microliter; mmol/L: millimoles per liter; mg/dL: milligrams per deciliter; g/dL: grams per deciliter; mL/min/1.73 m²: milliliters per minute normalized to a body surface area of 1.73 m²

Parameter	Value	Reference range	Interpretation
WBC (×10³/µL)	9.9	4.0-11.0	Within normal limits
Hemoglobin (g/dL)	12.2	13.5-17.5	Mild anemia
Hematocrit (%)	38.6	41-53	Mildly decreased
Platelets (×10³/µL)	255	150-400	Within normal limits
Sodium (mmol/L)	131-133	135-145	Mild hyponatremia
Potassium (mmol/L)	4.0-4.6	3.5-5.0	Within normal limits
Creatinine (mg/dL)	0.75-0.95	0.6-1.3	Normal renal function
eGFR (mL/min/1.73 m²)	>60	>60	Preserved renal function
BUN (mg/dL)	21-30	7-20	Mildly elevated
Albumin (g/dL)	2.8-3.4	3.5-5.0	Hypoalbuminemia
Calcium (mg/dL)	7.8-8.8	8.6-10.2	Mildly decreased
Phosphate (mg/dL)	1.9	2.5-4.5	Hypophosphatemia
Glucose (mg/dL)	122-167	70-99	Hyperglycemia
Lipase (U/L)	28	~11-82	Within normal limits

Initial imaging

**Figure 1:**
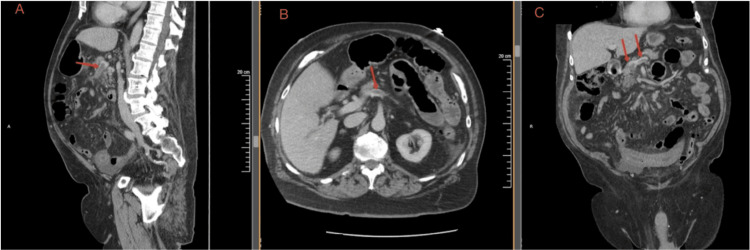
Contrast-enhanced computed tomography demonstrating superior mesenteric vein thrombosis (red arrows) Multiplanar contrast-enhanced computed tomography of the abdomen demonstrating thrombosis within the superior mesenteric venous system (arrows). (A) Sagittal reconstruction demonstrating a filling defect within the superior mesenteric vein near the portal confluence. (B) Axial image demonstrating the corresponding intraluminal filling defect within the superior mesenteric vein. (C) Coronal reconstruction further illustrating the thrombus within the superior mesenteric venous system. The portal vein appears patent. Associated bowel wall thickening and mesenteric edema are present without evidence of frank bowel infarction or pancreatic inflammation on the initial study.

Contrast-enhanced computed tomography (CT) of the abdomen and pelvis revealed non-occlusive thrombosis of the superior mesenteric vein extending toward the portal venous confluence, with additional non-occlusive thrombus noted within the splenic vein, as shown in Figure 1. The portal and hepatic veins were patent. Associated findings included bowel wall thickening of a loop of small bowel with adjacent mesenteric edema, consistent with veno-occlusive mesenteric ischemia. Mild ascites were also present. Notably, the pancreas appeared homogeneous and normal in contour, without evidence of pancreatic ductal dilation, focal pancreatic mass, or radiographic findings suggestive of pancreatitis. This interpretation was further supported by the absence of radiographic pancreatic inflammation and a normal lipase level at presentation.

The absence of pancreatitis was clinically relevant, as pancreatitis is a recognized benign precipitant of SVT. In the absence of inflammatory pancreatic findings or another obvious provoking intra-abdominal process, broader etiologic evaluation was pursued, including selective assessment for occult malignancy.

Further evaluation

Given the unusual splanchnic distribution of thrombosis, absence of cirrhosis or pancreatitis, and lack of an immediately apparent provoking factor on initial imaging, further evaluation for occult malignancy was pursued as part of individualized clinical decision-making. Positron emission tomography (PET)-CT performed on August 1, 2025, demonstrated no hypermetabolic lesions. Duplex ultrasonography confirmed preserved portal venous flow. Review of prior hematologic testing, obtained approximately eight months before presentation, demonstrated a heterozygous prothrombin G20210A mutation. Additional thrombophilia-related evaluation available for review was otherwise unrevealing, including negative testing for factor V Leiden, Janus kinase 2 (JAK2) V617F, calreticulin (CALR), myeloproliferative leukemia virus oncogene (MPL) mutations, breakpoint cluster region-Abelson fusion gene (BCR-ABL1), and paroxysmal nocturnal hemoglobinuria (PNH) (Table 2).

**Table 2:** Prior and admission-related hypercoagulability evaluation Hypercoagulability evaluation available for review, including previously documented heterozygous prothrombin (factor II) mutation and additional thrombophilia-related testing performed as part of the patient's hematologic evaluation. No evidence of myeloproliferative neoplasm, paroxysmal nocturnal hemoglobinuria, or another clearly established alternative thrombophilic disorder was identified. JAK2: Janus kinase 2; CALR: calreticulin; MPL: myeloproliferative leukemia virus oncogene; BCR-ABL1: breakpoint cluster region-Abelson murine leukemia viral oncogene fusion gene; LA: lupus anticoagulant; DRVVT: dilute Russell viper venom time; PTT: partial thromboplastin time

Test	Result	Interpretation
Prothrombin (factor II) mutation	Mutation detected	Consistent with inherited thrombophilia
Protein C activity (%)	85	Within normal range
Protein S activity (%)	88	Within normal range
JAK2 V617F mutation	Not detected	No evidence of myeloproliferative neoplasm
JAK2 exon 12 mutation	Not detected	No evidence of myeloproliferative neoplasm
CALR exon 9 mutation	Not detected	No evidence of myeloproliferative neoplasm
MPL exon 10 mutation	Not detected	No evidence of myeloproliferative neoplasm
BCR-ABL1 (%)	0.000	No evidence of chronic myelogenous leukemia
Paroxysmal nocturnal hemoglobinuria	Negative	No evidence of clonal hemolytic disorder
Baseline LA PTT (seconds)	37.2	Within reference range
DRVVT screen	48.0 (elevated)	Positive screening test
DRVVT screen ratio	1.33 (elevated)	Abnormal
DRVVT normalized ratio	1.09	Borderline
Thrombin time (seconds)	19.5	Within reference range

Lupus anticoagulant testing demonstrated an abnormal screening profile but did not establish a definitive diagnosis of antiphospholipid syndrome. These findings were interpreted cautiously in a clinical context, particularly given the absence of supporting clinical history and the lack of a clearly diagnostic, normalized confirmatory pattern. As such, they were considered potentially confounding but insufficient to independently explain the thrombotic event.

Management and follow-up

**Figure 2:**
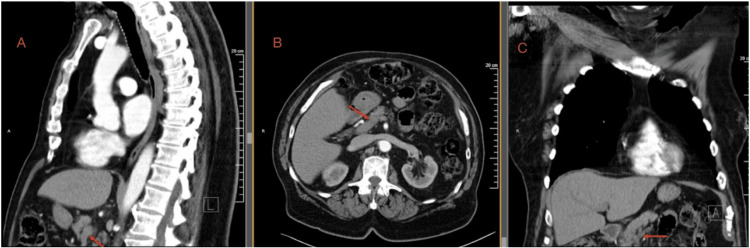
Follow-up contrast-enhanced computed tomography demonstrating the resolution of splanchnic venous thrombosis (red arrows) after anticoagulation Multiplanar contrast-enhanced computed tomography of the abdomen obtained three months after the initiation of apixaban therapy demonstrating the complete radiologic resolution of previously identified superior mesenteric and splenic vein thrombi. (A) Sagittal reconstruction demonstrating the normal opacification of the mesenteric venous system without residual filling defect. (B) Axial image demonstrating the patency of the superior mesenteric vein at the prior site of thrombosis (arrow). (C) Coronal reconstruction confirming the restoration of normal venous flow within the mesenteric venous confluence.

The patient was initially treated with inpatient therapeutic unfractionated heparin, with anticoagulation monitored using anti-factor Xa levels during hospitalization. After remaining clinically stable without bleeding or radiographic concern for thrombus progression, he was transitioned to apixaban 5 mg twice daily for continued outpatient anticoagulation and counseled regarding long-term therapy. His abdominal symptoms resolved, and he remained clinically stable. Repeat CT imaging of the chest and abdomen in November 2025 demonstrated complete resolution of the superior mesenteric and splenic vein thrombosis, as shown in Figure 2.

An incidental right upper-lobe pulmonary nodule decreased in size from 1 cm to 7 mm and was interpreted as benign. Laboratory testing at follow-up showed stable renal and hepatic function. At the six-month follow-up, the patient remained asymptomatic with no bleeding complications or recurrent thrombosis. A summary of the patient's clinical presentation, diagnostic evaluation, and management timeline is provided in Table 3.

**Table 3:** Timeline of clinical presentation, diagnostic evaluation, and management Timeline of the patient's presentation, diagnostic evaluation, anticoagulation course, and follow-up. CT: computed tomography; PET: positron emission tomography; SMV: superior mesenteric vein; anti-Xa: anti-factor Xa

Date	Event	Key findings
July 2025	Initial presentation	Several days of vague abdominal discomfort and early satiety
July 2025	Physical examination and laboratory evaluation	Mild epigastric tenderness; mild anemia without leukocytosis or thrombocytopenia; renal function preserved; lipase within normal limits
July 2025	Contrast-enhanced CT of the abdomen/pelvis	Non-occlusive SMV thrombosis extending into the splenic vein with associated bowel wall thickening and mesenteric edema; portal vein patent
Prior to July 2025 (reviewed during admission)	Hypercoagulability evaluation	Previously documented heterozygous prothrombin G20210A mutation and additional thrombophilia-related testing available for review; no clearly established alternative thrombophilic disorder identified
August 2025	PET-CT	No hypermetabolic lesions identified
August 2025	Duplex ultrasonography	Preserved portal venous flow
July-August 2025	Inpatient anticoagulation and transition to outpatient therapy	Initial therapeutic unfractionated heparin with anti-Xa monitoring, followed by transition to apixaban 5 mg twice daily
November 2025	Follow-up CT of the chest/abdomen	Complete resolution of SMV and splenic vein thromboses
November 2025	Laboratory follow-up	Stable renal and hepatic function
May 2026	Clinical follow-up	No recurrent thrombosis or bleeding

Following diagnosis, the patient was managed with therapeutic anticoagulation and close inpatient monitoring prior to transition to long-term oral therapy. Anti-factor Xa levels remained within or near the therapeutic range during this period, without evidence of bleeding complications or clinical deterioration. Inpatient anticoagulation monitoring is summarized in Table 4.

**Table 4:** Inpatient anticoagulation monitoring (unfractionated heparin phase) Anti-Xa: anti-factor Xa; IU/mL: international units per milliliter

Measurement	Value (IU/mL)
Anti-Xa level 1	0.52
Anti-Xa level 2	0.34
Anti-Xa level 3	0.94

After completing the initial inpatient anticoagulation phase without clinical deterioration or bleeding complications, the patient was transitioned to standard therapeutic apixaban dosing for continued outpatient treatment of venous thrombosis. Renal function remained preserved throughout treatment, and no bleeding complications were observed during follow-up.

## Discussion

Concurrent thrombosis of the superior mesenteric and splenic veins represents an uncommon SVT pattern, particularly in the absence of cirrhosis, malignancy, or inflammatory intra-abdominal disease [9]. When secondary causes are excluded, inherited thrombophilias should be strongly considered. In the absence of pancreatitis or another clear provoking intra-abdominal process, isolated or dominant superior mesenteric vein thrombosis may prompt additional evaluation for occult pancreatic or other intra-abdominal pathology, depending on clinical context.

The prothrombin G20210A mutation is an established inherited thrombophilia caused by a G-to-A substitution at nucleotide 20210 in the 3′ untranslated region of the prothrombin gene. This variant has been associated with elevated circulating prothrombin levels and enhanced thrombin generation, thereby increasing susceptibility to venous thromboembolism [6]. However, heterozygous carriers are generally considered to have a moderate thrombotic risk, and many remain asymptomatic throughout life. 

In this context, the present case likely reflects a multifactorial thrombotic event in which inherited susceptibility interacted with age-related and cardiometabolic risk factors, lowering the threshold for thrombosis [10,11]. The occurrence of symptomatic SVT in the eighth decade of life, despite the likely lifelong presence of the mutation, supports a model in which genetic predisposition alone may be insufficient for clinical expression without additional acquired contributors. In addition to age, the patient's baseline cardiometabolic profile, including diabetes, hypertension, hyperlipidemia, and mildly atherogenic lipid parameters, may also have contributed to an overall prothrombotic milieu.

Accurate diagnosis and follow-up of SVT rely heavily on imaging. Contrast-enhanced CT remains the preferred initial modality because of its high sensitivity for mesenteric and splenic venous thrombosis and its ability to evaluate bowel viability [8]. Given that unprovoked venous thrombosis may represent an early manifestation of occult malignancy, PET-CT was used as complementary imaging to exclude an underlying cancer [12]. Serial imaging subsequently demonstrated complete recanalization, highlighting the role of repeat imaging in monitoring therapeutic response.

Systemic anticoagulation is the cornerstone of SVT management. While vitamin K antagonists (VKAs) were historically favored, growing evidence supports the use of DOACs, such as apixaban, in non-cirrhotic, non-malignant SVT [4,13,14]. Observational data suggest comparable efficacy with favorable safety profiles [14]. The favorable radiologic and clinical course observed in this patient is consistent with the growing observational literature describing DOAC use in selected cases of non-cirrhotic, non-malignant SVT.

A comprehensive hematologic workup is paramount for distinguishing between inherited and acquired causes of SVT [4,15]. The "triple-negative" status for myeloproliferative neoplasm (MPN) drivers, specifically the absence of JAK2 V617F, CALR, and MPL mutations, was crucial in this case. By effectively ruling out occult MPN, which frequently manifests as splanchnic thrombosis [16], the heterozygous prothrombin G20210A mutation likely represented one contributing thrombophilic factor within a broader multifactorial thrombotic context. This diagnostic clarity is essential for determining the duration of anticoagulation and the necessity of long-term hematologic surveillance [4,15].

**Figure 3:**
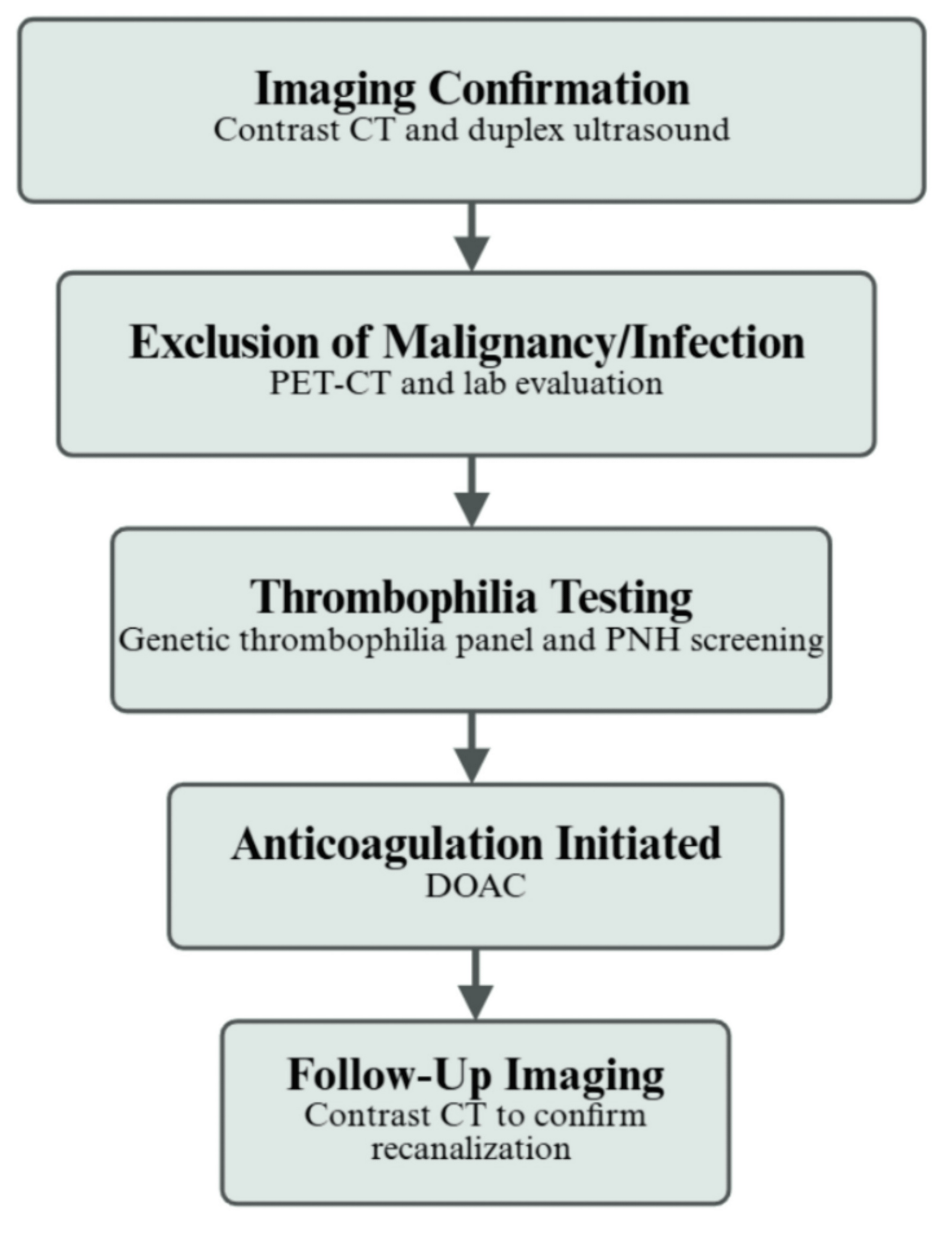
Conceptual workflow for the evaluation of non-cirrhotic splanchnic venous thrombosis Conceptual diagram outlining a structured diagnostic approach to splanchnic venous thrombosis, including imaging confirmation, exclusion of malignancy and inflammatory causes, thrombophilia testing, anticoagulation, and follow-up imaging. This figure is an original work of the authors, created using BioRender, without the use of artificial intelligence (AI). CT: computed tomography; PET: positron emission tomography; PNH: paroxysmal nocturnal hemoglobinuria; DOAC: direct oral anticoagulant

This case illustrates how inherited thrombophilia may manifest later in life when superimposed upon age-related vascular vulnerability. It underscores the importance of structured evaluation in non-cirrhotic SVT and supports the consideration of DOACs in appropriately selected patients. A structured diagnostic approach to non-cirrhotic SVT is outlined in Figure 3. Larger prospective studies remain necessary to clarify optimal treatment duration and comparative efficacy among anticoagulant strategies.

Limitations

This report describes a single patient and therefore cannot establish causality or determine comparative efficacy among anticoagulation strategies. Although complete radiographic resolution was observed following treatment with apixaban, the observational nature of this case precludes a definitive conclusion regarding superiority over VKAs or other DOACs. Follow-up was limited to six months, and longer-term outcomes, including recurrence risk and recanalization durability, remain unknown. In addition, although a comprehensive hypercoagulable and malignancy evaluation was performed, plasma prothrombin levels were not serially measured, and subtle, transient provoking factors, such as dehydration, may not have been fully quantifiable. Finally, imaging confirmation relied on cross-sectional modalities without invasive venographic correlation, though this reflects standard clinical practice. Despite these limitations, the case contributes clinically relevant insight into the presentation and management of non-cirrhotic, thrombophilia-associated SVT. In addition, the thrombotic event in this patient was likely multifactorial rather than attributable to inherited thrombophilia alone, as advanced age, cardiometabolic comorbidities, and possibly unmeasured transient contributors may also have lowered the threshold for thrombosis. Borderline lupus anticoagulant-related findings were present but were not definitively diagnostic and therefore could not be conclusively interpreted as causal or incidental.

## Conclusions

This case describes concurrent superior mesenteric and splenic vein thrombosis in an older adult with a heterozygous prothrombin G20210A mutation and no clear alternative provoking intra-abdominal cause. Complete radiologic resolution following anticoagulation highlights a favorable clinical course in this individual patient. This report adds to the growing body of observational experience by describing the use of DOACs in selected cases of non-cirrhotic SVT. Careful etiologic evaluation and individualized follow-up remain important for guiding management.
